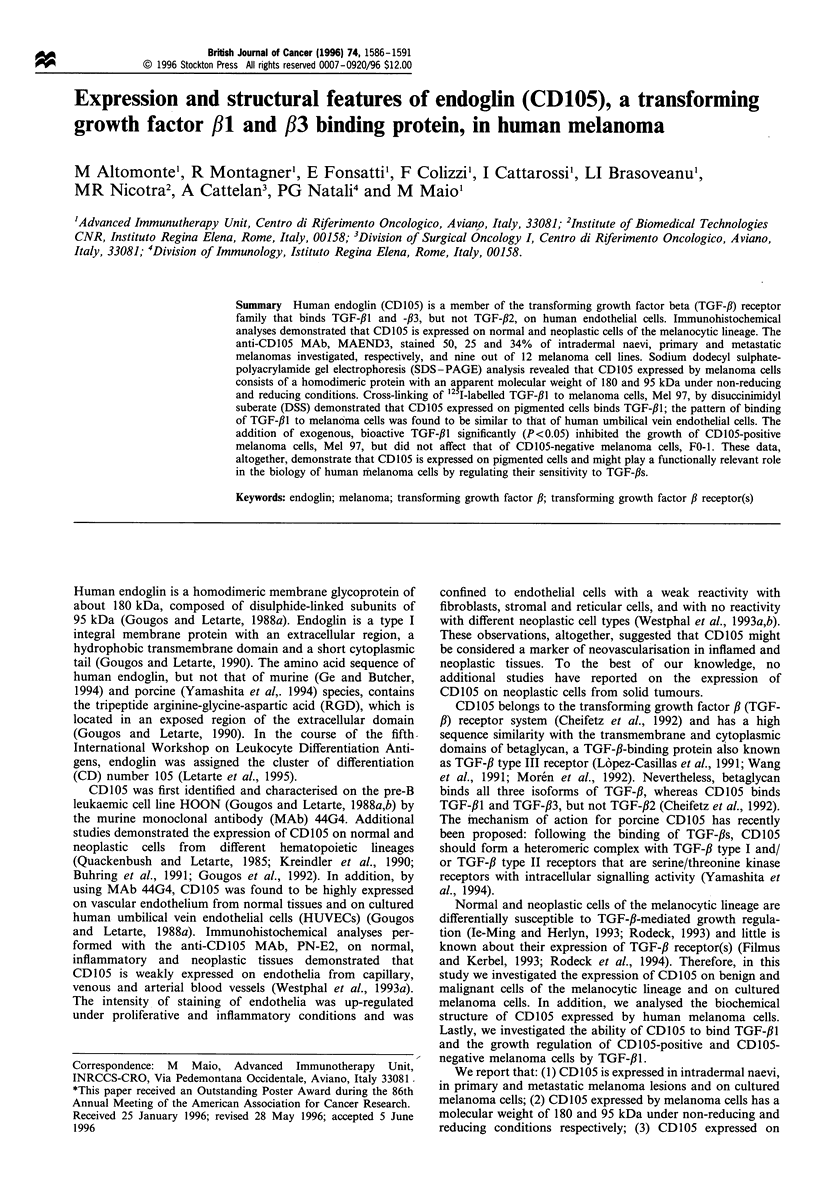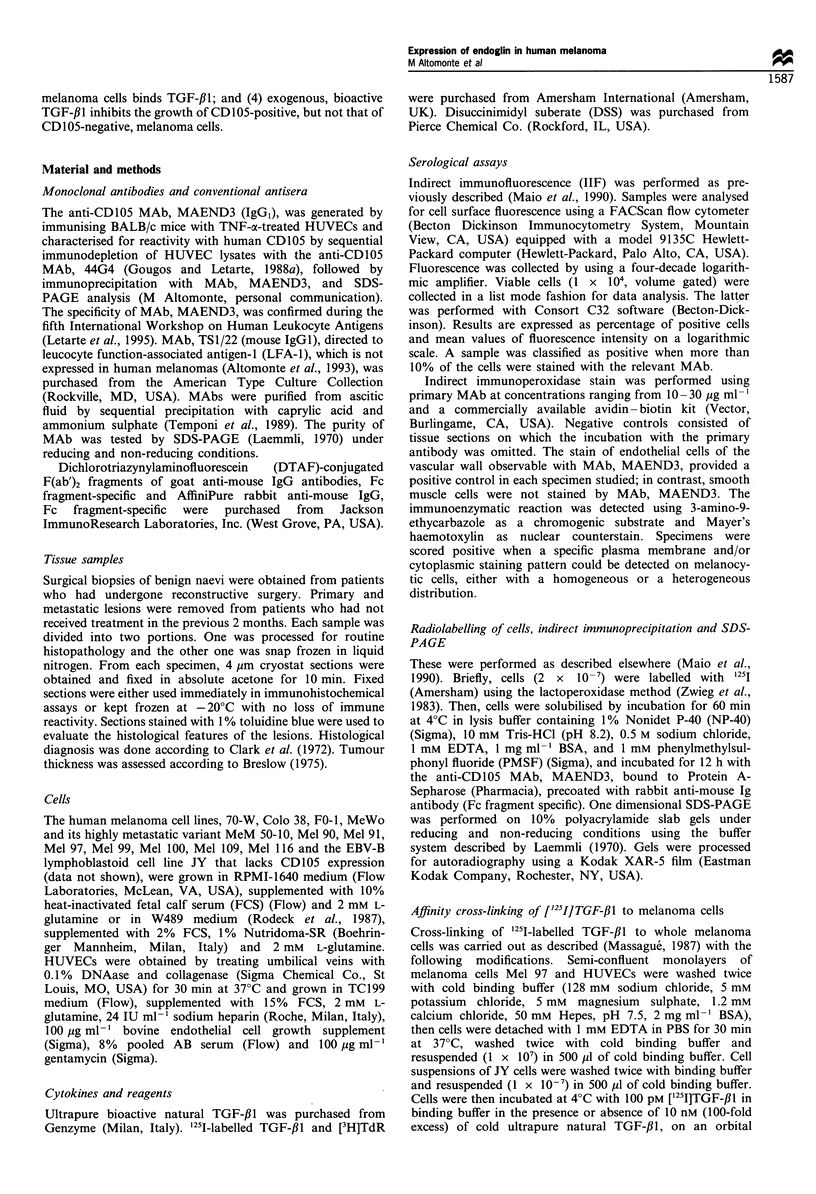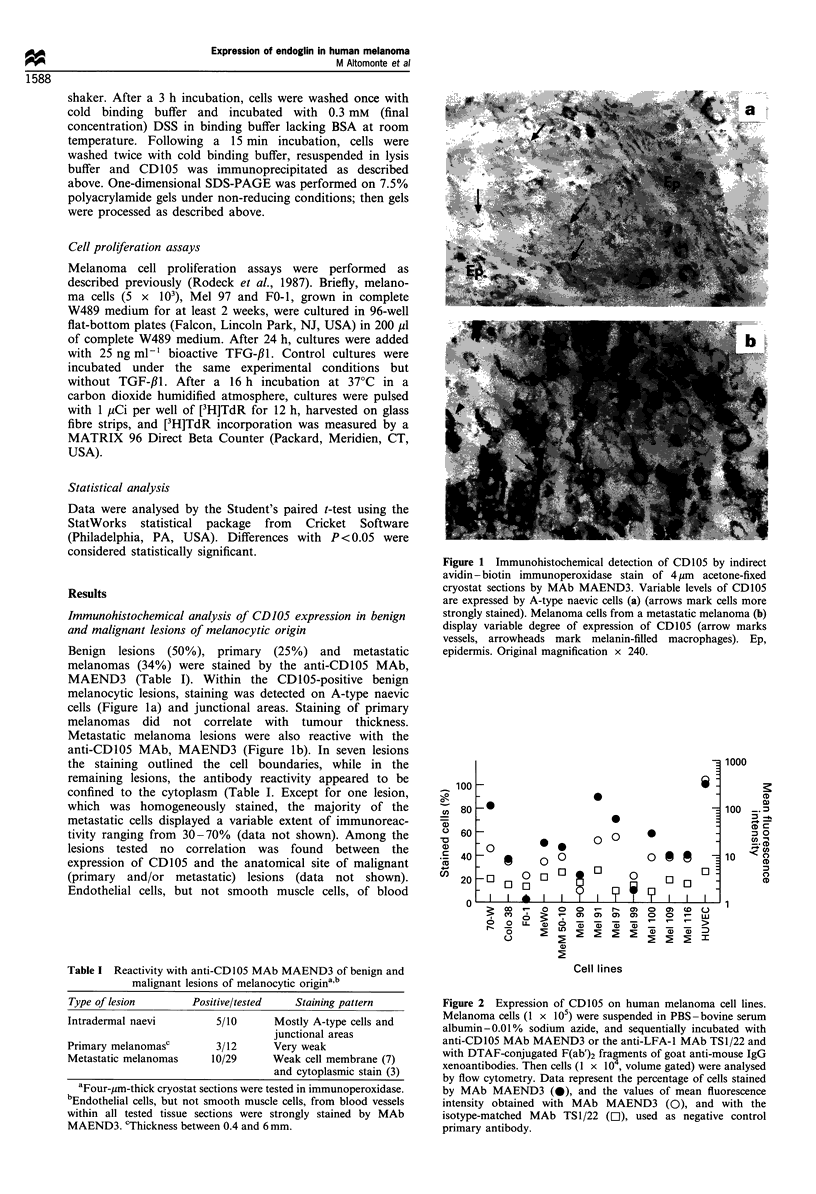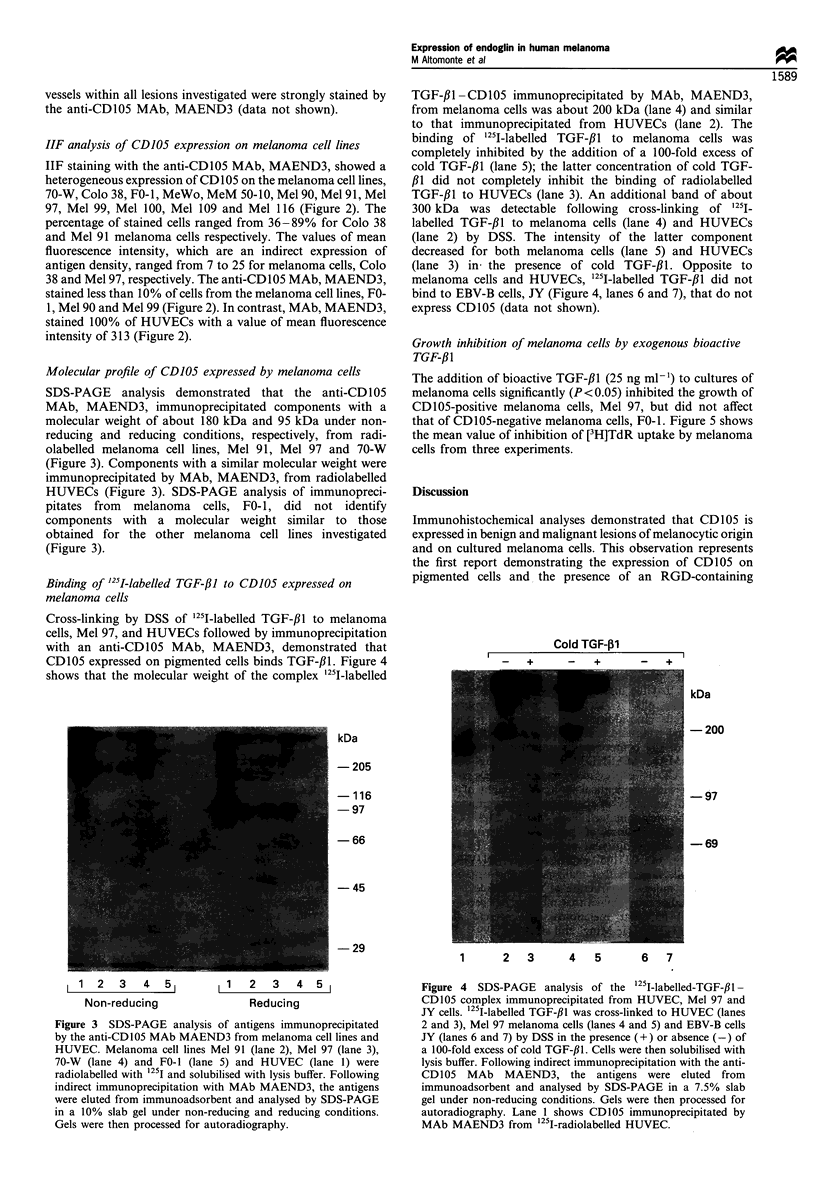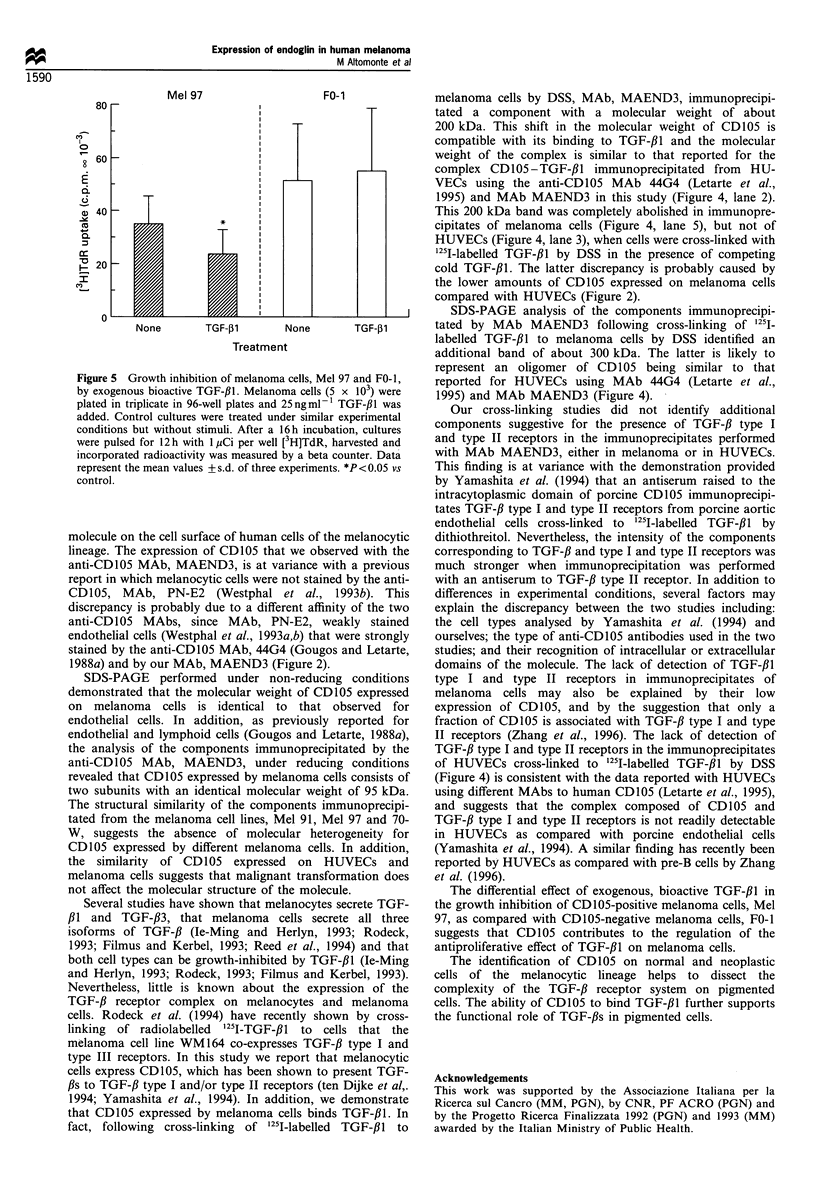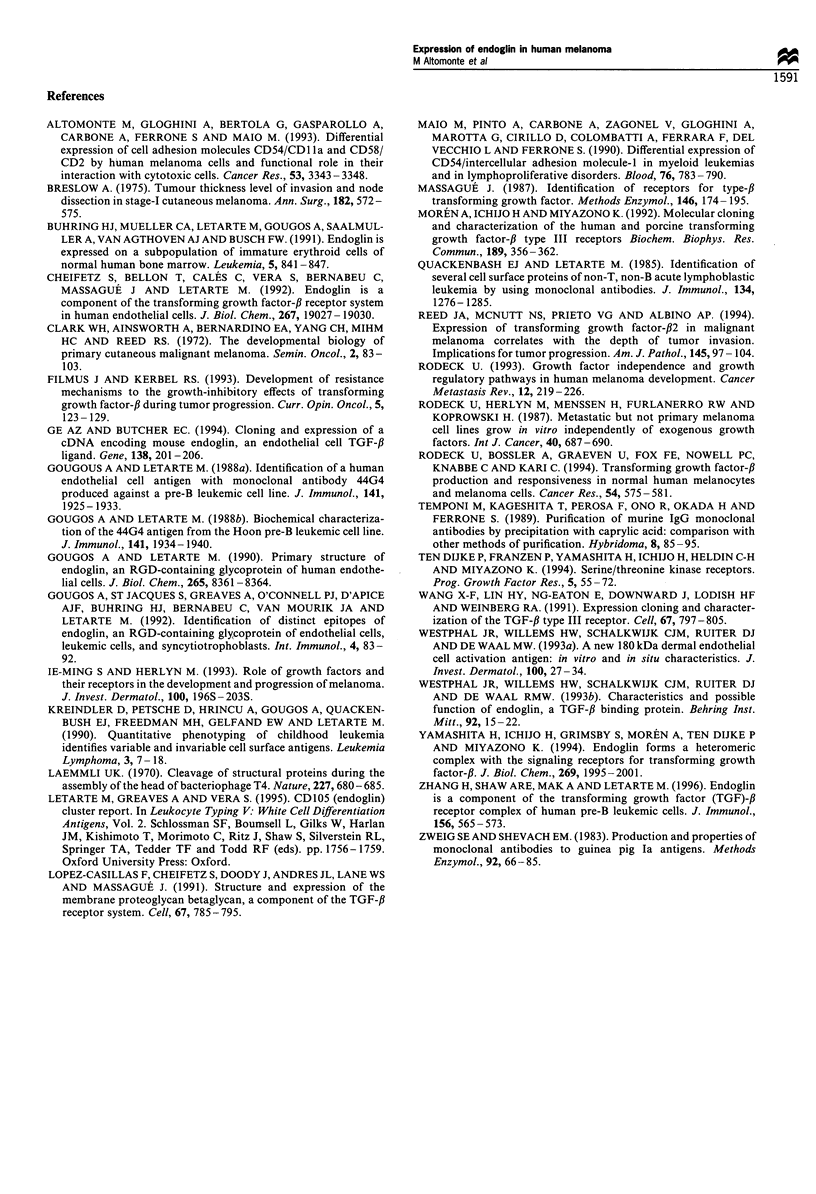# Expression and structural features of endoglin (CD105), a transforming growth factor beta1 and beta3 binding protein, in human melanoma.

**DOI:** 10.1038/bjc.1996.593

**Published:** 1996-11

**Authors:** M. Altomonte, R. Montagner, E. Fonsatti, F. Colizzi, I. Cattarossi, L. I. Brasoveanu, M. R. Nicotra, A. Cattelan, P. G. Natali, M. Maio

**Affiliations:** Advanced Immunutherapy Unit, Centro di Riferimento Oncologico, Aviano, Italy.

## Abstract

**Images:**


British Journal of Cancer (1996) 74, 1586-1591
© 1996 Stockton Press All rights reserved 0007-0920/96 $12.00

### Expression and structural features of endoglin (CD105), a transforming growth factor β1 and β3 binding protein, in human melanoma

M Altomonte[1], R Montagner[1], E Fonsatti[1], F Colizzi[1], I Cattarossi[1], LI Brasoveanu[1], MR Nicotra[2], A Cattelan[3], PG Natali[4] and M Maio[1]

[1]*Advanced Immunutherapy Unit, Centro di Riferimento Oncologico, Aviano, Italy, 33081;* [2]*Institute of Biomedical Technologies CNR, Instituto Regina Elena, Rome, Italy, 00158;* [3]*Division of Surgical Oncology I, Centro di Riferimento Oncologico, Aviano, Italy, 33081;* [4]*Division of Immunology, Istituto Regina Elena, Rome, Italy, 00158.*

**Summary** Human endoglin (CD105) is a member of the transforming growth factor beta (TGF-β) receptor family that binds TGF-β1 and -β3, but not TGF-β2, on human endothelial cells. Immunohistochemical analyses demonstrated that CD105 is expressed on normal and neoplastic cells of the melanocytic lineage. The anti-CD105 MAb, MAEND3, stained 50, 25 and 34% of intradermal naevi, primary and metastatic melanomas investigated, respectively, and nine out of 12 melanoma cell lines. Sodium dodecyl sulphate-polyacrylamide gel electrophoresis (SDS-PAGE) analysis revealed that CD105 expressed by melanoma cells consists of a homodimeric protein with an apparent molecular weight of 180 and 95 kDa under non-reducing and reducing conditions. Cross-linking of $^{125}$I-labelled TGF-β1 to melanoma cells, Mel 97, by disuccinimidyl suberate (DSS) demonstrated that CD105 expressed on pigmented cells binds TGF-β1; the pattern of binding of TGF-β1 to melanoma cells was found to be similar to that of human umbilical vein endothelial cells. The addition of exogenous, bioactive TGF-β1 significantly ($P<0.05$) inhibited the growth of CD105-positive melanoma cells, Mel 97, but did not affect that of CD105-negative melanoma cells, F0-1. These data, altogether, demonstrate that CD105 is expressed on pigmented cells and might play a functionally relevant role in the biology of human melanoma cells by regulating their sensitivity to TGF-βs.

**Keywords:** endoglin; melanoma; transforming growth factor β; transforming growth factor β receptor(s)

Human endoglin is a homodimeric membrane glycoprotein of about 180 kDa, composed of disulphide-linked subunits of 95 kDa (Gougos and Letarte, 1988*a*). Endoglin is a type I integral membrane protein with an extracellular region, a hydrophobic transmembrane domain and a short cytoplasmic tail (Gougos and Letarte, 1990). The amino acid sequence of human endoglin, but not that of murine (Ge and Butcher, 1994) and porcine (Yamashita *et al*,. 1994) species, contains the tripeptide arginine-glycine-aspartic acid (RGD), which is located in an exposed region of the extracellular domain (Gougos and Letarte, 1990). In the course of the fifth International Workshop on Leukocyte Differentiation Antigens, endoglin was assigned the cluster of differentiation (CD) number 105 (Letarte *et al.*, 1995).

CD105 was first identified and characterised on the pre-B leukaemic cell line HOON (Gougos and Letarte, 1988*a,b*) by the murine monoclonal antibody (MAb) 44G4. Additional studies demonstrated the expression of CD105 on normal and neoplastic cells from different hematopoietic lineages (Quackenbush and Letarte, 1985; Kreindler *et al.*, 1990; Buhring *et al.*, 1991; Gougos *et al.*, 1992). In addition, by using MAb 44G4, CD105 was found to be highly expressed on vascular endothelium from normal tissues and on cultured human umbilical vein endothelial cells (HUVECs) (Gougos and Letarte, 1988*a*). Immunohistochemical analyses performed with the anti-CD105 MAb, PN-E2, on normal, inflammatory and neoplastic tissues demonstrated that CD105 is weakly expressed on endothelia from capillary, venous and arterial blood vessels (Westphal *et al.*, 1993*a*). The intensity of staining of endothelia was up-regulated under proliferative and inflammatory conditions and was confined to endothelial cells with a weak reactivity with fibroblasts, stromal and reticular cells, and with no reactivity with different neoplastic cell types (Westphal *et al.*, 1993*a,b*). These observations, altogether, suggested that CD105 might be considered a marker of neovascularisation in inflamed and neoplastic tissues. To the best of our knowledge, no additional studies have reported on the expression of CD105 on neoplastic cells from solid tumours.

CD105 belongs to the transforming growth factor β (TGF-β) receptor system (Cheifetz *et al.*, 1992) and has a high sequence similarity with the transmembrane and cytoplasmic domains of betaglycan, a TGF-β-binding protein also known as TGF-β type III receptor (Lòpez-Casillas *et al.*, 1991; Wang *et al.*, 1991; Morén *et al.*, 1992). Nevertheless, betaglycan binds all three isoforms of TGF-β, whereas CD105 binds TGF-β1 and TGF-β3, but not TGF-β2 (Cheifetz *et al.*, 1992). The mechanism of action for porcine CD105 has recently been proposed: following the binding of TGF-βs, CD105 should form a heteromeric complex with TGF-β type I and/or TGF-β type II receptors that are serine/threonine kinase receptors with intracellular signalling activity (Yamashita *et al.*, 1994).

Normal and neoplastic cells of the melanocytic lineage are differentially susceptible to TGF-β-mediated growth regulation (Ie-Ming and Herlyn, 1993; Rodeck, 1993) and little is known about their expression of TGF-β receptor(s) (Filmus and Kerbel, 1993; Rodeck *et al.*, 1994). Therefore, in this study we investigated the expression of CD105 on benign and malignant cells of the melanocytic lineage and on cultured melanoma cells. In addition, we analysed the biochemical structure of CD105 expressed by human melanoma cells. Lastly, we investigated the ability of CD105 to bind TGF-β1 and the growth regulation of CD105-positive and CD105-negative melanoma cells by TGF-β1.

We report that: (1) CD105 is expressed in intradermal naevi, in primary and metastatic melanoma lesions and on cultured melanoma cells; (2) CD105 expressed by melanoma cells has a molecular weight of 180 and 95 kDa under non-reducing and reducing conditions respectively; (3) CD105 expressed on

Correspondence: M Maio, Advanced Immunotherapy Unit, INRCCS-CRO, Via Pedemontana Occidentale, Aviano, Italy 33081.
*This paper received an Outstanding Poster Award during the 86th Annual Meeting of the American Association for Cancer Research.
Received 25 January 1996; revised 28 May 1996; accepted 5 June 1996

melanoma cells binds TGF-$\beta$1; and (4) exogenous, bioactive TGF-$\beta$1 inhibits the growth of CD105-positive, but not that of CD105-negative, melanoma cells.

### Material and methods

*Monoclonal antibodies and conventional antisera*

The anti-CD105 MAb, MAEND3 ($IgG_1$), was generated by immunising BALB/c mice with TNF-$\alpha$-treated HUVECs and characterised for reactivity with human CD105 by sequential immunodepletion of HUVEC lysates with the anti-CD105 MAb, 44G4 (Gougos and Letarte, 1988*a*), followed by immunoprecipitation with MAb, MAEND3, and SDS-PAGE analysis (M Altomonte, personal communication). The specificity of MAb, MAEND3, was confirmed during the fifth International Workshop on Human Leukocyte Antigens (Letarte *et al.*, 1995). MAb, TS1/22 (mouse IgG1), directed to leucocyte function-associated antigen-1 (LFA-1), which is not expressed in human melanomas (Altomonte *et al.*, 1993), was purchased from the American Type Culture Collection (Rockville, MD, USA). MAbs were purified from ascitic fluid by sequential precipitation with caprylic acid and ammonium sulphate (Temponi *et al.*, 1989). The purity of MAb was tested by SDS-PAGE (Laemmli, 1970) under reducing and non-reducing conditions.

Dichlorotriazynylaminofluorescein (DTAF)-conjugated $F(ab')_2$ fragments of goat anti-mouse IgG antibodies, Fc fragment-specific and AffiniPure rabbit anti-mouse IgG, Fc fragment-specific were purchased from Jackson ImmunoResearch Laboratories, Inc. (West Grove, PA, USA).

*Tissue samples*

Surgical biopsies of benign naevi were obtained from patients who had undergone reconstructive surgery. Primary and metastatic lesions were removed from patients who had not received treatment in the previous 2 months. Each sample was divided into two portions. One was processed for routine histopathology and the other one was snap frozen in liquid nitrogen. From each specimen, 4 $\mu$m cryostat sections were obtained and fixed in absolute acetone for 10 min. Fixed sections were either used immediately in immunohistochemical assays or kept frozen at $-20$°C with no loss of immune reactivity. Sections stained with 1% toluidine blue were used to evaluate the histological features of the lesions. Histological diagnosis was done according to Clark *et al.* (1972). Tumour thickness was assessed according to Breslow (1975).

*Cells*

The human melanoma cell lines, 70-W, Colo 38, F0-1, MeWo and its highly metastatic variant MeM 50-10, Mel 90, Mel 91, Mel 97, Mel 99, Mel 100, Mel 109, Mel 116 and the EBV-B lymphoblastoid cell line JY that lacks CD105 expression (data not shown), were grown in RPMI-1640 medium (Flow Laboratories, McLean, VA, USA), supplemented with 10% heat-inactivated fetal calf serum (FCS) (Flow) and 2 mM L-glutamine or in W489 medium (Rodeck *et al.*, 1987), supplemented with 2% FCS, 1% Nutridoma-SR (Boehringer Mannheim, Milan, Italy) and 2 mM L-glutamine. HUVECs were obtained by treating umbilical veins with 0.1% DNAase and collagenase (Sigma Chemical Co., St Louis, MO, USA) for 30 min at 37°C and grown in TC199 medium (Flow), supplemented with 15% FCS, 2 mM L-glutamine, 24 IU $ml^{-1}$ sodium heparin (Roche, Milan, Italy), 100 $\mu g\ ml^{-1}$ bovine endothelial cell growth supplement (Sigma), 8% pooled AB serum (Flow) and 100 $\mu g\ ml^{-1}$ gentamycin (Sigma).

*Cytokines and reagents*

Ultrapure bioactive natural TGF-$\beta$1 was purchased from Genzyme (Milan, Italy). $^{125}$I-labelled TGF-$\beta$1 and [$^3$H]TdR were purchased from Amersham International (Amersham, UK). Disuccinimidyl suberate (DSS) was purchased from Pierce Chemical Co. (Rockford, IL, USA).

*Serological assays*

Indirect immunofluorescence (IIF) was performed as previously described (Maio et al., 1990). Samples were analysed for cell surface fluorescence using a FACScan flow cytometer (Becton Dickinson Immunocytometry System, Mountain View, CA, USA) equipped with a model 9135C Hewlett-Packard computer (Hewlett-Packard, Palo Alto, CA, USA). Fluorescence was collected by using a four-decade logarithmic amplifier. Viable cells ($1 \times 10^4$, volume gated) were collected in a list mode fashion for data analysis. The latter was performed with Consort C32 software (Becton-Dickinson). Results are expressed as percentage of positive cells and mean values of fluorescence intensity on a logarithmic scale. A sample was classified as positive when more than 10% of the cells were stained with the relevant MAb.

Indirect immunoperoxidase stain was performed using primary MAb at concentrations ranging from 10–30 $\mu g\ ml^{-1}$ and a commercially available avidin–biotin kit (Vector, Burlingame, CA, USA). Negative controls consisted of tissue sections on which the incubation with the primary antibody was omitted. The stain of endothelial cells of the vascular wall observable with MAb, MAEND3, provided a positive control in each specimen studied; in contrast, smooth muscle cells were not stained by MAb, MAEND3. The immunoenzymatic reaction was detected using 3-amino-9-ethycarbazole as a chromogenic substrate and Mayer's haemotoxylin as nuclear counterstain. Specimens were scored positive when a specific plasma membrane and/or cytoplasmic staining pattern could be detected on melanocytic cells, either with a homogeneous or a heterogeneous distribution.

*Radiolabelling of cells, indirect immunoprecipitation and SDS-PAGE*

These were performed as described elsewhere (Maio *et al.*, 1990). Briefly, cells ($2 \times 10^{-7}$) were labelled with $^{125}$I (Amersham) using the lactoperoxidase method (Zwieg *et al.*, 1983). Then, cells were solubilised by incubation for 60 min at 4°C in lysis buffer containing 1% Nonidet P-40 (NP-40) (Sigma), 10 mM Tris-HCl (pH 8.2), 0.5 M sodium chloride, 1 mM EDTA, 1 mg $ml^{-1}$ BSA, and 1 mM phenylmethylsulphonyl fluoride (PMSF) (Sigma), and incubated for 12 h with the anti-CD105 MAb, MAEND3, bound to Protein A-Sepharose (Pharmacia), precoated with rabbit anti-mouse Ig antibody (Fc fragment specific). One dimensional SDS-PAGE was performed on 10% polyacrylamide slab gels under reducing and non-reducing conditions using the buffer system described by Laemmli (1970). Gels were processed for autoradiography using a Kodak XAR-5 film (Eastman Kodak Company, Rochester, NY, USA).

*Affinity cross-linking of [$^{125}$I]TGF-$\beta$1* to melanoma cells

Cross-linking of $^{125}$I-labelled TGF-$\beta$1 to whole melanoma cells was carried out as described (Massagué, 1987) with the following modifications. Semi-confluent monolayers of melanoma cells Mel 97 and HUVECs were washed twice with cold binding buffer (128 mM sodium chloride, 5 mM potassium chloride, 5 mM magnesium sulphate, 1.2 mM calcium chloride, 50 mM Hepes, pH 7.5, 2 mg $ml^{-1}$ BSA), then cells were detached with 1 mM EDTA in PBS for 30 min at 37°C, washed twice with cold binding buffer and resuspended ($1 \times 10^7$) in 500 $\mu$l of cold binding buffer. Cell suspensions of JY cells were washed twice with binding buffer and resuspended ($1 \times 10^{-7}$) in 500 $\mu$l of cold binding buffer. Cells were then incubated at 4°C with 100 pM [$^{125}$I]TGF-$\beta$1 in binding buffer in the presence or absence of 10 nM (100-fold excess) of cold ultrapure natural TGF-$\beta$1, on an orbital shaker. After a 3 h incubation, cells were washed once with cold binding buffer and incubated with 0.3 mM (final concentration) DSS in binding buffer lacking BSA at room temperature. Following a 15 min incubation, cells were washed twice with cold binding buffer, resuspended in lysis buffer and CD105 was immunoprecipitated as described above. One-dimensional SDS-PAGE was performed on 7.5% polyacrylamide gels under non-reducing conditions; then gels were processed as described above.

*Cell proliferation assays*

Melanoma cell proliferation assays were performed as described previously (Rodeck *et al.*, 1987). Briefly, melanoma cells (5 × $10^3$), Mel 97 and F0-1, grown in complete W489 medium for at least 2 weeks, were cultured in 96-well flat-bottom plates (Falcon, Lincoln Park, NJ, USA) in 200 $\mu$l of complete W489 medium. After 24 h, cultures were added with 25 ng $ml^{-1}$ bioactive TFG-$\beta$1. Control cultures were incubated under the same experimental conditions but without TGF-$\beta$1. After a 16 h incubation at 37°C in a carbon dioxide humidified atmosphere, cultures were pulsed with 1 $\mu$Ci per well of [$^3$H]TdR for 12 h, harvested on glass fibre strips, and [$^3$H]TdR incorporation was measured by a MATRIX 96 Direct Beta Counter (Packard, Meridien, CT, USA).

*Statistical analysis*

Data were analysed by the Student's paired *t*-test using the StatWorks statistical package from Cricket Software (Philadelphia, PA, USA). Differences with $P<0.05$ were considered statistically significant.

### Results

*Immunohistochemical analysis of CD105 expression in benign and malignant lesions of melanocytic origin*

Benign lesions (50%), primary (25%) and metastatic melanomas (34%) were stained by the anti-CD105 MAb, MAEND3 (Table I). Within the CD105-positive benign melanocytic lesions, staining was detected on A-type naevic cells (Figure 1a) and junctional areas. Staining of primary melanomas did not correlate with tumour thickness. Metastatic melanoma lesions were also reactive with the anti-CD105 MAb, MAEND3 (Figure 1b). In seven lesions the staining outlined the cell boundaries, while in the remaining lesions, the antibody reactivity appeared to be confined to the cytoplasm (Table I. Except for one lesion, which was homogeneously stained, the majority of the metastatic cells displayed a variable extent of immunoreactivity ranging from 30–70% (data not shown). Among the lesions tested no correlation was found between the expression of CD105 and the anatomical site of malignant (primary and/or metastatic) lesions (data not shown). Endothelial cells, but not smooth muscle cells, of blood

**Table I** Reactivity with anti-CD105 MAb MAEND3 of benign and malignant lesions of melanocytic origin[a,b]

| *Type of lesion* | *Positive/tested* | *Staining pattern* |
|---|---|---|
| Intradermal naevi | 5/10 | Mostly A-type cells and junctional areas |
| Primary melanomas[c] | 3/12 | Very weak |
| Metastatic melanomas | 10/29 | Weak cell membrane (7) and cytoplasmic stain (3) |

[a]Four-$\mu$m-thick cryostat sections were tested in immunoperoxidase. [b]Endothelial cells, but not smooth muscle cells, from blood vessels within all tested tissue sections were strongly stained by MAb MAEND3. [c]Thickness between 0.4 and 6 mm.

**Figure 1** Immunohistochemical detection of CD105 by indirect avidin–biotin immunoperoxidase stain of 4 $\mu$m acetone-fixed cryostat sections by MAb MAEND3. Variable levels of CD105 are expressed by A-type naevic cells (**a**) (arrows mark cells more strongly stained). Melanoma cells from a metastatic melanoma (**b**) display variable degree of expression of CD105 (arrow marks vessels, arrowheads mark melanin-filled macrophages). Ep, epidermis. Original magnification × 240.

**Figure 2** Expression of CD105 on human melanoma cell lines. Melanoma cells (1 × $10^5$) were suspended in PBS–bovine serum albumin–0.01% sodium azide, and sequentially incubated with anti-CD105 MAb MAEND3 or the anti-LFA-1 MAb TS1/22 and with DTAF-conjugated F(ab')$_2$ fragments of goat anti-mouse IgG xenoantibodies. Then cells (1 × $10^4$, volume gated) were analysed by flow cytometry. Data represent the percentage of cells stained by MAb MAEND3 (●), and the values of mean fluorescence intensity obtained with MAb MAEND3 (○), and with the isotype-matched MAb TS1/22 (□), used as negative control primary antibody.

vessels within all lesions investigated were strongly stained by the anti-CD105 MAb, MAEND3 (data not shown).

*IIF analysis of CD105 expression on melanoma cell lines*

IIF staining with the anti-CD105 MAb, MAEND3, showed a heterogeneous expression of CD105 on the melanoma cell lines, 70-W, Colo 38, F0-1, MeWo, MeM 50-10, Mel 90, Mel 91, Mel 97, Mel 99, Mel 100, Mel 109 and Mel 116 (Figure 2). The percentage of stained cells ranged from 36–89% for Colo 38 and Mel 91 melanoma cells respectively. The values of mean fluorescence intensity, which are an indirect expression of antigen density, ranged from 7 to 25 for melanoma cells, Colo 38 and Mel 97, respectively. The anti-CD105 MAb, MAEND3, stained less than 10% of cells from the melanoma cell lines, F0-1, Mel 90 and Mel 99 (Figure 2). In contrast, MAb, MAEND3, stained 100% of HUVECs with a value of mean fluorescence intensity of 313 (Figure 2).

*Molecular profile of CD105 expressed by melanoma cells*

SDS-PAGE analysis demonstrated that the anti-CD105 MAb, MAEND3, immunoprecipitated components with a molecular weight of about 180 kDa and 95 kDa under non-reducing and reducing conditions, respectively, from radiolabelled melanoma cell lines, Mel 91, Mel 97 and 70-W (Figure 3). Components with a similar molecular weight were immunoprecipitated by MAb, MAEND3, from radiolabelled HUVECs (Figure 3). SDS-PAGE analysis of immunoprecipitates from melanoma cells, F0-1, did not identify components with a molecular weight similar to those obtained for the other melanoma cell lines investigated (Figure 3).

*Binding of $^{125}$I-labelled TGF-$\beta$1 to CD105 expressed on melanoma cells*

Cross-linking by DSS of $^{125}$I-labelled TGF-$\beta$1 to melanoma cells, Mel 97, and HUVECs followed by immunoprecipitation with an anti-CD105 MAb, MAEND3, demonstrated that CD105 expressed on pigmented cells binds TGF-$\beta$1. Figure 4 shows that the molecular weight of the complex $^{125}$I-labelled TGF-$\beta$1–CD105 immunoprecipitated by MAb, MAEND3, from melanoma cells was about 200 kDa (lane 4) and similar to that immunoprecipitated from HUVECs (lane 2). The binding of $^{125}$I-labelled TGF-$\beta$1 to melanoma cells was completely inhibited by the addition of a 100-fold excess of cold TGF-$\beta$1 (lane 5); the latter concentration of cold TGF-$\beta$1 did not completely inhibit the binding of radiolabelled TGF-$\beta$1 to HUVECs (lane 3). An additional band of about 300 kDa was detectable following cross-linking of $^{125}$I-labelled TGF-$\beta$1 to melanoma cells (lane 4) and HUVECs (lane 2) by DSS. The intensity of the latter component decreased for both melanoma cells (lane 5) and HUVECs (lane 3) in the presence of cold TGF-$\beta$1. Opposite to melanoma cells and HUVECs, $^{125}$I-labelled TGF-$\beta$1 did not bind to EBV-B cells, JY (Figure 4, lanes 6 and 7), that do not express CD105 (data not shown).

*Growth inhibition of melanoma cells by exogenous bioactive TGF-$\beta$1*

The addition of bioactive TGF-$\beta$1 (25 ng ml$^{-1}$) to cultures of melanoma cells significantly ($P<0.05$) inhibited the growth of CD105-positive melanoma cells, Mel 97, but did not affect that of CD105-negative melanoma cells, F0-1. Figure 5 shows the mean value of inhibition of [$^3$H]TdR uptake by melanoma cells from three experiments.

### Discussion

Immunohistochemical analyses demonstrated that CD105 is expressed in benign and malignant lesions of melanocytic origin and on cultured melanoma cells. This observation represents the first report demonstrating the expression of CD105 on pigmented cells and the presence of an RGD-containing

**Figure 3** SDS-PAGE analysis of antigens immunoprecipitated by the anti-CD105 MAb MAEND3 from melanoma cell lines and HUVEC. Melanoma cell lines Mel 91 (lane 2), Mel 97 (lane 3), 70-W (lane 4) and F0-1 (lane 5) and HUVEC (lane 1) were radiolabelled with $^{125}$I and solubilised with lysis buffer. Following indirect immunoprecipitation with MAb MAEND3, the antigens were eluted from immunoadsorbent and analysed by SDS-PAGE in a 10% slab gel under non-reducing and reducing conditions. Gels were then processed for autoradiography.

**Figure 4** SDS-PAGE analysis of the $^{125}$I-labelled-TGF-$\beta$1–CD105 complex immunoprecipitated from HUVEC, Mel 97 and JY cells. $^{125}$I-labelled TGF-$\beta$1 was cross-linked to HUVEC (lanes 2 and 3), Mel 97 melanoma cells (lanes 4 and 5) and EBV-B cells JY (lanes 6 and 7) by DSS in the presence (+) or absence (–) of a 100-fold excess of cold TGF-$\beta$1. Cells were then solubilised with lysis buffer. Following indirect immunoprecipitation with the anti-CD105 MAb MAEND3, the antigens were eluted from immunoadsorbent and analysed by SDS-PAGE in a 7.5% slab gel under non-reducing conditions. Gels were then processed for autoradiography. Lane 1 shows CD105 immunoprecipitated by MAb MAEND3 from $^{125}$I-radiolabelled HUVEC.

**Figure 5** Growth inhibition of melanoma cells, Mel 97 and F0-1, by exogenous bioactive TGF-$\beta$1. Melanoma cells ($5 \times 10^3$) were plated in triplicate in 96-well plates and 25 ng ml$^{-1}$ TGF-$\beta$1 was added. Control cultures were treated under similar experimental conditions but without stimuli. After a 16 h incubation, cultures were pulsed for 12 h with 1 $\mu$Ci per well [$^3$H]TdR, harvested and incorporated radioactivity was measured by a beta counter. Data represent the mean values $\pm$s.d. of three experiments. *$P<0.05$ *vs* control.

molecule on the cell surface of human cells of the melanocytic lineage. The expression of CD105 that we observed with the anti-CD105 MAb, MAEND3, is at variance with a previous report in which melanocytic cells were not stained by the anti-CD105, MAb, PN-E2 (Westphal *et al.*, 1993*b*). This discrepancy is probably due to a different affinity of the two anti-CD105 MAbs, since MAb, PN-E2, weakly stained endothelial cells (Westphal *et al.*, 1993*a,b*) that were strongly stained by the anti-CD105 MAb, 44G4 (Gougos and Letarte, 1988*a*) and by our MAb, MAEND3 (Figure 2).

SDS-PAGE performed under non-reducing conditions demonstrated that the molecular weight of CD105 expressed on melanoma cells is identical to that observed for endothelial cells. In addition, as previously reported for endothelial and lymphoid cells (Gougos and Letarte, 1988*a*), the analysis of the components immunoprecipitated by the anti-CD105 MAb, MAEND3, under reducing conditions revealed that CD105 expressed by melanoma cells consists of two subunits with an identical molecular weight of 95 kDa. The structural similarity of the components immunoprecipitated from the melanoma cell lines, Mel 91, Mel 97 and 70-W, suggests the absence of molecular heterogeneity for CD105 expressed by different melanoma cells. In addition, the similarity of CD105 expressed on HUVECs and melanoma cells suggests that malignant transformation does not affect the molecular structure of the molecule.

Several studies have shown that melanocytes secrete TGF-$\beta$1 and TGF-$\beta$3, that melanoma cells secrete all three isoforms of TGF-$\beta$ (Ie-Ming and Herlyn, 1993; Rodeck, 1993; Filmus and Kerbel, 1993; Reed *et al.*, 1994) and that both cell types can be growth-inhibited by TGF-$\beta$1 (Ie-Ming and Herlyn, 1993; Rodeck, 1993; Filmus and Kerbel, 1993). Nevertheless, little is known about the expression of the TGF-$\beta$ receptor complex on melanocytes and melanoma cells. Rodeck *et al.* (1994) have recently shown by cross-linking of radiolabelled $^{125}$I-TGF-$\beta$1 to cells that the melanoma cell line WM164 co-expresses TGF-$\beta$ type I and type III receptors. In this study we report that melanocytic cells express CD105, which has been shown to present TGF-$\beta$s to TGF-$\beta$ type I and/or type II receptors (ten Dijke *et al*,. 1994; Yamashita *et al.*, 1994). In addition, we demonstrate that CD105 expressed by melanoma cells binds TGF-$\beta$1. In fact, following cross-linking of $^{125}$I-labelled TGF-$\beta$1 to melanoma cells by DSS, MAb, MAEND3, immunoprecipitated a component with a molecular weight of about 200 kDa. This shift in the molecular weight of CD105 is compatible with its binding to TGF-$\beta$1 and the molecular weight of the complex is similar to that reported for the complex CD105–TGF-$\beta$1 immunoprecipitated from HUVECs using the anti-CD105 MAb 44G4 (Letarte *et al.*, 1995) and MAb MAEND3 in this study (Figure 4, lane 2). This 200 kDa band was completely abolished in immunoprecipitates of melanoma cells (Figure 4, lane 5), but not of HUVECs (Figure 4, lane 3), when cells were cross-linked with $^{125}$I-labelled TGF-$\beta$1 by DSS in the presence of competing cold TGF-$\beta$1. The latter discrepancy is probably caused by the lower amounts of CD105 expressed on melanoma cells compared with HUVECs (Figure 2).

SDS-PAGE analysis of the components immunoprecipitated by MAb MAEND3 following cross-linking of $^{125}$I-labelled TGF-$\beta$1 to melanoma cells by DSS identified an additional band of about 300 kDa. The latter is likely to represent an oligomer of CD105 being similar to that reported for HUVECs using MAb 44G4 (Letarte *et al.*, 1995) and MAb MAEND3 (Figure 4).

Our cross-linking studies did not identify additional components suggestive for the presence of TGF-$\beta$ type I and type II receptors in the immunoprecipitates performed with MAb MAEND3, either in melanoma or in HUVECs. This finding is at variance with the demonstration provided by Yamashita *et al.* (1994) that an antiserum raised to the intracytoplasmic domain of porcine CD105 immunoprecipitates TGF-$\beta$ type I and type II receptors from porcine aortic endothelial cells cross-linked to $^{125}$I-labelled TGF-$\beta$1 by dithiothreitol. Nevertheless, the intensity of the components corresponding to TGF-$\beta$ and type I and type II receptors was much stronger when immunoprecipitation was performed with an antiserum to TGF-$\beta$ type II receptor. In addition to differences in experimental conditions, several factors may explain the discrepancy between the two studies including: the cell types analysed by Yamashita *et al.* (1994) and ourselves; the type of anti-CD105 antibodies used in the two studies; and their recognition of intracellular or extracellular domains of the molecule. The lack of detection of TGF-$\beta$1 type I and type II receptors in immunoprecipitates of melanoma cells may also be explained by their low expression of CD105, and by the suggestion that only a fraction of CD105 is associated with TGF-$\beta$ type I and type II receptors (Zhang *et al.*, 1996). The lack of detection of TGF-$\beta$ type I and type II receptors in the immunoprecipitates of HUVECs cross-linked to $^{125}$I-labelled TGF-$\beta$1 by DSS (Figure 4) is consistent with the data reported with HUVECs using different MAbs to human CD105 (Letarte *et al.*, 1995), and suggests that the complex composed of CD105 and TGF-$\beta$ type I and type II receptors is not readily detectable in HUVECs as compared with porcine endothelial cells (Yamashita *et al.*, 1994). A similar finding has recently been reported by HUVECs as compared with pre-B cells by Zhang *et al.* (1996).

The differential effect of exogenous, bioactive TGF-$\beta$1 in the growth inhibition of CD105-positive melanoma cells, Mel 97, as compared with CD105-negative melanoma cells, F0-1 suggests that CD105 contributes to the regulation of the antiproliferative effect of TGF-$\beta$1 on melanoma cells.

The identification of CD105 on normal and neoplastic cells of the melanocytic lineage helps to dissect the complexity of the TGF-$\beta$ receptor system on pigmented cells. The ability of CD105 to bind TGF-$\beta$1 further supports the functional role of TGF-$\beta$s in pigmented cells.

**Acknowledgements**
This work was supported by the Associazione Italiana per la Ricerca sul Cancro (MM, PGN), by CNR, PF ACRO (PGN) and by the Progetto Ricerca Finalizzata 1992 (PGN) and 1993 (MM) awarded by the Italian Ministry of Public Health.

### References

ALTOMONTE M, GLOGHINI A, BERTOLA G, GASPAROLLO A, CARBONE A, FERRONE S AND MAIO M. (1993). Differential expression of cell adhesion molecules CD54/CD11a and CD58/CD2 by human melanoma cells and functional role in their interaction with cytotoxic cells. *Cancer Res.*, **53**, 3343–3348.

BRESLOW A. (1975). Tumour thickness level of invasion and node dissection in stage-I cutaneous melanoma. *Ann. Surg.*, **182**, 572–575.

BUHRING HJ, MUELLER CA, LETARTE M, GOUGOS A, SAALMULLER A, VAN AGTHOVEN AJ AND BUSCH FW. (1991). Endoglin is expressed on a subpopulation of immature erythroid cells of normal human bone marrow. *Leukemia*, **5**, 841–847.

CHEIFETZ S, BELLON T, CALÉS C, VERA S, BERNABEU C, MASSAGUÉ J AND LETARTE M. (1992). Endoglin is a component of the transforming growth factor-β receptor system in human endothelial cells. *J. Biol. Chem.*, **267**, 19027–19030.

CLARK WH, AINSWORTH A, BERNARDINO EA, YANG CH, MIHM HC AND REED RS. (1972). The developmental biology of primary cutaneous malignant melanoma. *Semin. Oncol.*, **2**, 83–103.

FILMUS J AND KERBEL RS. (1993). Development of resistance mechanisms to the growth-inhibitory effects of transforming growth factor-β during tumor progression. *Curr. Opin. Oncol.*, **5**, 123–129.

GE AZ AND BUTCHER EC. (1994). Cloning and expression of a cDNA encoding mouse endoglin, an endothelial cell TGF-β ligand. *Gene*, **138**, 201–206.

GOUGOUS A AND LETARTE M. (1988*a*). Identification of a human endothelial cell antigen with monoclonal antibody 44G4 produced against a pre-B leukemic cell line. *J. Immunol.*, **141**, 1925–1933.

GOUGOS A AND LETARTE M. (1988*b*). Biochemical characterization of the 44G4 antigen from the Hoon pre-B leukemic cell line. *J. Immunol.*, **141**, 1934–1940.

GOUGOS A AND LETARTE M. (1990). Primary structure of endoglin, an RGD-containing glycoprotein of human endothelial cells. *J. Biol. Chem.*, **265**, 8361–8364.

GOUGOS A, ST JACQUES S, GREAVES A, O'CONNELL PJ, D'APICE AJF, BUHRING HJ, BERNABEU C, VAN MOURIK JA AND LETARTE M. (1992). Identification of distinct epitopes of endoglin, an RGD-containing glycoprotein of endothelial cells, leukemic cells, and syncytiotrophoblasts. *Int. Immunol.*, **4**, 83–92.

IE-MING S AND HERLYN M. (1993). Role of growth factors and their receptors in the development and progression of melanoma. *J. Invest. Dermatol.*, **100**, 196S–203S.

KREINDLER D, PETSCHE D, HRINCU A, GOUGOS A, QUACKENBUSH EJ, FREEDMAN MH, GELFAND EW AND LETARTE M. (1990). Quantitative phenotyping of childhood leukemia identifies variable and invariable cell surface antigens. *Leukemia Lymphoma*, **3**, 7–18.

LAEMMLI UK. (1970). Cleavage of structural proteins during the assembly of the head of bacteriophage T4. *Nature*, **227**, 680–685.

LETARTE M, GREAVES A AND VERA S. (1995). CD105 (endoglin) cluster report. In *Leukocyte Typing V: White Cell Differentiation Antigens*, Vol. 2. Schlossman SF, Boumsell L, Gilks W, Harlan JM, Kishimoto T, Morimoto C, Ritz J, Shaw S, Silverstein RL, Springer TA, Tedder TF and Todd RF (eds). pp. 1756–1759. Oxford University Press: Oxford.

LOPEZ-CASILLAS F, CHEIFETZ S, DOODY J, ANDRES JL, LANE WS AND MASSAGUÉ J. (1991). Structure and expression of the membrane proteoglycan betaglycan, a component of the TGF-β receptor system. *Cell*, **67**, 785–795.

MAIO M, PINTO A, CARBONE A, ZAGONEL V, GLOGHINI A, MAROTTA G, CIRILLO D, COLOMBATTI A, FERRARA F, DEL VECCHIO L AND FERRONE S. (1990). Differential expression of CD54/intercellular adhesion molecule-1 in myeloid leukemias and in lymphoproliferative disorders. *Blood*, **76**, 783–790.

MASSAGUÉ J. (1987). Identification of receptors for type-β transforming growth factor. *Methods Enzymol.*, **146**, 174–195.

MORÉN A, ICHIJO H AND MIYAZONO K. (1992). Molecular cloning and characterization of the human and porcine transforming growth factor-β type III receptors *Biochem. Biophys. Res. Commun.*, **189**, 356–362.

QUACKENBASH EJ AND LETARTE M. (1985). Identification of several cell surface proteins of non-T, non-B acute lymphoblastic leukemia by using monoclonal antibodies. *J. Immunol.*, **134**, 1276–1285.

REED JA, MCNUTT NS, PRIETO VG AND ALBINO AP. (1994). Expression of transforming growth factor-β2 in malignant melanoma correlates with the depth of tumor invasion. Implications for tumor progression. *Am. J. Pathol.*, **145**, 97–104.

RODECK U. (1993). Growth factor independence and growth regulatory pathways in human melanoma development. *Cancer Metastasis Rev.*, **12**, 219–226.

RODECK U, HERLYN M, MENSSEN H, FURLANERRO RW AND KOPROWSKI H. (1987). Metastatic but not primary melanoma cell lines grow *in vitro* independently of exogenous growth factors. *Int J. Cancer*, **40**, 687–690.

RODECK U, BOSSLER A, GRAEVEN U, FOX FE, NOWELL PC, KNABBE C AND KARI C. (1994). Transforming growth factor-β production and responsiveness in normal human melanocytes and melanoma cells. *Cancer Res.*, **54**, 575–581.

TEMPONI M, KAGESHITA T, PEROSA F, ONO R, OKADA H AND FERRONE S. (1989). Purification of murine IgG monoclonal antibodies by precipitation with caprylic acid: comparison with other methods of purification. *Hybridoma*, **8**, 85–95.

TEN DIJKE P, FRANZEN P, YAMASHITA H, ICHIJO H, HELDIN C-H AND MIYAZONO K. (1994). Serine/threonine kinase receptors. *Prog. Growth Factor Res.*, **5**, 55–72.

WANG X-F, LIN HY, NG-EATON E, DOWNWARD J, LODISH HF AND WEINBERG RA. (1991). Expression cloning and characterization of the TGF-β type III receptor. *Cell*, **67**, 797–805.

WESTPHAL JR, WILLEMS HW, SCHALKWIJK CJM, RUITER DJ AND DE WAAL MW. (1993*a*). A new 180 kDa dermal endothelial cell activation antigen: *in vitro* and *in situ* characteristics. *J. Invest. Dermatol.*, **100**, 27–34.

WESTPHAL JR, WILLEMS HW, SCHALKWIJK CJM, RUITER DJ AND DE WAAL RMW. (1993*b*). Characteristics and possible function of endoglin, a TGF-β binding protein. *Behring Inst. Mitt.*, **92**, 15–22.

YAMASHITA H, ICHIJO H, GRIMSBY S, MORÉN A, TEN DIJKE P AND MIYAZONO K. (1994). Endoglin forms a heteromeric complex with the signaling receptors for transforming growth factor-β. *J. Biol. Chem.*, **269**, 1995–2001.

ZHANG H, SHAW ARE, MAK A AND LETARTE M. (1996). Endoglin is a component of the transforming growth factor (TGF)-β receptor complex of human pre-B leukemic cells. *J. Immunol.*, **156**, 565–573.

ZWEIG SE AND SHEVACH EM. (1983). Production and properties of monoclonal antibodies to guinea pig Ia antigens. *Methods Enzymol.*, **92**, 66–85.